# Identification of Colorectal Cancer-Related RNA Markers from Whole Blood Using Integrated Bioinformatics Analysis

**DOI:** 10.3390/ijms262311625

**Published:** 2025-11-30

**Authors:** Jin Han, Jung Chul Na, Tae Il Kim, Jae Myun Lee, Jong Koo Kim, Jae Jun Park, Jaemee Jung, Hyeyoung Lee

**Affiliations:** 1 Department of Biomedical Laboratory Science, College of Software and Digital Healthcare Convergence, Yonsei University Mirae Campus, Wonju 26493, Republic of Korea; kristenlovemom@gmail.com (J.H.); jungchul.na@handok.com (J.C.N.);; 2 Division of Gastroenterology, Department of Internal Medicine, Yonsei University College of Medicine, Seoul 03722, Republic of Korea; 3 Department of Family Medicine, Wonju College of Medicine, Yonsei University, Wonju 26426, Republic of Korea; 4 Department of Microbiology and Immunology, Institute for Immunology and Immunological Diseases, Yonsei University College of Medicine, Seoul 03722, Republic of Korea; 5 INOGENIX Inc., Chuncheon 24232, Republic of Korea

**Keywords:** colorectal cancer, whole blood, liquid biopsy, RNA-seq, circulating transcripts, biomarker discovery, RT-qPCR, early detection

## Abstract

Despite advances in blood-based screening tests for colorectal cancer (CRC), most existing assays focus on DNA-based biomarkers, which predominantly reflect tumor-derived fragments released at later disease stages. In contrast, whole-blood transcriptomic profiling can capture systemic immune responses and tumor–host interactions, offering a complementary strategy for earlier disease detection. However, clinically validated whole-blood transcriptomic signatures remain limited. Here, we investigated a whole-blood RNA-based biomarker discovery strategy by integrating multi-cohort transcriptomic resources. Public GEO datasets (GSE164191 and GSE11545) were harmonized and analyzed, yielding 956 differentially expressed genes (DEGs). Multi-layer biological filtering incorporating PPI networks, transcription factors, CRC-related GWAS variants, whole-blood eQTL signals, DigSeE, and CoReCG disease associations refined these to 375 high-confidence transcripts (WB-PADs). In parallel, RNA-seq analysis of a Korean cohort (10 CRC vs. 10 controls) identified 217 DEGs (WB-K). Cross-dataset convergence highlighted seven overlapping transcripts, and five candidates (*DLG5*, *CD177*, *SH2D1B*, *NQO2*, and *KRT73*) were selected for validation. RT-qPCR in an independent clinical cohort (106 CRC and 123 healthy controls) confirmed four transcripts with significant discriminatory ability. A multivariable logistic regression model derived from the five-transcript signature achieved an AUC of 0.952 (95% CI 0.884–1.000), with sensitivities of 0.889 and 0.667 at fixed specificities of 90% and 95%, respectively, demonstrating strong applicability for screening-relevant thresholds. Notably, the model retained high accuracy in early-stage CRC (Stage I–II: AUC 0.929, 95% CI 0.868–0.989). Overall, this study provides a robust analytic framework for reproducible whole-blood RNA biomarker discovery and establishes a multi-gene signature with promising translational potential for minimally invasive and early CRC detection.

## 1. Introduction

Liquid biopsy has emerged as a promising platform for non-invasive cancer diagnostics, enabling real-time monitoring of tumor dynamics. In colorectal cancer (CRC), most blood-based studies have focused on circulating tumor DNA (ctDNA), methylation markers, or circulating tumor cells (CTCs) [1,2]. While these assays provide valuable tumor-specific information, they primarily reflect tumor-derived fragments released at later stages of disease, limiting their sensitivity for early detection. In contrast, RNA-based profiling captures dynamic transcriptional activity from both tumor cells and the host immune system [3]. This complementary information may reveal systemic immune responses and tumor–host interactions that precede detectable DNA release, offering opportunities for earlier CRC detection.

RNA molecules exhibit remarkable structural and functional diversity. Six major types have been identified, including messenger RNAs (mRNAs), microRNAs (miRNAs), long non-coding RNAs (lncRNAs), circular RNAs (circRNAs), transfer RNA-derived fragments (tRFs), and PIWI-interacting RNAs (piRNAs). Recent studies have shown that each class plays distinct roles in tumorigenesis, immune modulation, and cancer progression. For instance, miRNAs and lncRNAs serve as crucial post-transcriptional regulators, while circRNAs often function as competing endogenous RNAs that modulate miRNA availability. These RNA species have therefore emerged as promising liquid biopsy biomarkers for colorectal cancer diagnosis and monitoring.

CRC progression is influenced not only by tumor-intrinsic alterations but also by extensive immune remodeling. Chronic inflammation is a well-recognized driver of carcinogenesis, with innate immune cells playing pivotal roles. Tumor-associated neutrophils promote angiogenesis and immunosuppression, while macrophages frequently acquire M2-like phenotypes that facilitate tumor progression and metastasis. Although these events occur within the tumor microenvironment, systemic alterations can also be observed in the circulation, such as elevated neutrophil-to-lymphocyte ratios and altered monocyte subsets [4]. These systemic signatures suggest that whole-blood transcriptomic profiling may provide a peripheral view of tumor–host interactions and immune remodeling. Unlike ctDNA assays, which primarily detect tumor-derived fragments released at later stages of disease, whole-blood transcriptomic profiling captures dynamic immune and inflammatory responses that emerge during early tumor–host interactions. Moreover, compared with PBMC-based assays that exclude granulocytes and platelets, whole-blood RNA encompasses all circulating cell types, thereby providing a more comprehensive view of systemic immune remodeling.

Peripheral blood can be analyzed in several formats, including plasma, serum, peripheral blood mononuclear cells (PBMCs), and whole blood (WB). Unlike plasma cfRNA or PBMC RNA, WB integrates signals across all leukocyte subsets, together with erythrocytes, platelets, and plasma components. WB transcriptomic profiling therefore offers a more comprehensive representation of systemic immune activity and may increase the likelihood of detecting clinically relevant RNA signatures [5].

Several studies have investigated WB-derived RNA as a source of cancer biomarkers. Park et al. identified ten tumor-associated circulating transcripts (TACTs) with diagnostic value in breast cancer [6]. Han et al. applied the same panel to CRC and reported promising, but inconsistent, performance across markers [7]. Kim et al. further examined WB RNA expression along the adenoma–carcinoma sequence (ACS), identifying *IFI27*, *DEFA4*, *MPO*, and *CD177* as stage-associated transcripts [8]. Collectively, these studies indicate the feasibility of WB transcriptomic analysis, while also highlighting the need for systematic strategies to identify and validate CRC-specific signatures across independent cohorts.

In the present study, an integrative framework was developed to reduce cohort-specific bias and to enhance the reproducibility of biomarker discovery. Public whole-blood transcriptome datasets were combined with newly generated RNA-seq data from Korean clinical samples, followed by independent RT-qPCR validation. Candidate transcripts were further evaluated against multiple CRC-related resources—including hub gene networks, transcription factors, GWAS, eQTL, DigSeE, and CoReCG—to ensure biological relevance. This multi-layer validation strategy increased confidence that the selected transcripts are robustly associated with CRC pathogenesis and provides a basis for developing non-invasive multi-marker panels for early detection and disease monitoring in CRC. We hypothesized that whole-blood transcriptomic profiling can capture immune-related transcriptional alterations reflecting tumor–host interactions, which may precede detectable tumor DNA release. This study aimed to integrate multi-cohort transcriptomic data to identify robust circulating RNA signatures associated with colorectal cancer.

**Figure 1:**
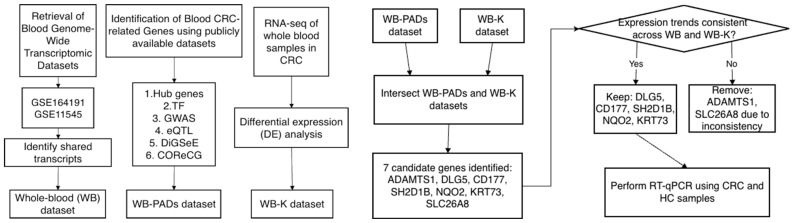
Graphical summary of the integrative analysis and clinical validation workflow.

A schematic overview of the experimental design and multi-cohort analytical workflow is presented in Figure 1.

## 2. Results

### 2.1. Selection and Preprocessing of GEO Whole-Blood Datasets

Peripheral blood gene expression datasets were retrieved from the Gene Expression Omnibus (GEO) database by searching with the keywords ‘Colorectal cancer’ and ‘Blood’. By manual inspection, dataset filtration was carried out based on two criteria: (1) transcriptome data from whole-blood samples and (2) availability of comparisons between CRC patients before any treatment and healthy controls. GSE164191 and GSE11545 datasets satisfying the above criteria were used to identify CRC-related genes. GSE164191 is a blood transcript data set containing 59 colorectal cancer samples and 62 normal controls. GSE11545 contains numerous cancer patient samples, among which we selected 9 colorectal cancer patient blood samples and 9 normal control group blood samples for analysis.

The GSE164191 dataset initially contained 54,700 probes. To minimize noise and false positives, 30% of probes with the lowest standard deviation across samples, and an additional 30% of probes with the lowest mean expression values among the remaining, were excluded. Similarly, from the 32,878 probes in GSE11545, 10% of low-variance probes and 10% of the remaining low-expression probes were removed. After filtering and annotation (Entrez ID), a total of 13,797 and 13,321 genes were retained from GSE164191 and GSE11545, respectively.

A total of 8966 genes were shared between the two datasets. To correct for inter-study variation, batch effects were normalized using the ComBat method, which combines a linear modeling framework with an empirical Bayesian approach. Principal component analysis (PCA) demonstrated a substantial reduction in platform-driven separation after correction (Figure S1). The two batch-corrected datasets were subsequently integrated to construct a comprehensive transcriptomic dataset derived from CRC-related whole-blood samples, encompassing expression profiles for 8966 genes. This integrated dataset was hereafter referred to as the WB dataset. Based on differential expression analysis, a total of 956 differentially expressed genes (DEGs) were identified between the CRC and control groups, applying a false discovery rate (FDR)-adjusted *p*-value < 0.05 and an absolute log2 fold-change (|log_2_FC|) > 0.5.

### 2.2. Construction of the WB-PADs Dataset

To identify robust and biologically relevant whole-blood (WB) transcripts associated with colorectal cancer (CRC), a multi-layer filtering strategy was applied to the 956 DEGs obtained from the integrated WB dataset. The rationale of this step was to prioritize genes supported by independent lines of biological or clinical evidence—such as protein interaction networks, transcriptional regulation, genetic associations, and curated CRC databases—thereby increasing confidence that the selected transcripts are functional and disease-relevant rather than dataset-specific artifacts.

First, a protein–protein interaction (PPI) network was constructed using the STRING database to capture genes with central roles in molecular signaling networks. A total of 956 DEGs were projected onto the network, and 132 genes with more than 10 connections (nodes) were identified as hub genes, designated as the WB-Hub dataset.

Next, to determine key transcriptional regulators, DEGs were cross-referenced with the Human Transcription Factors (TFs) Database (http://humantfs.ccbr.utoronto.ca/index.php (accessed on 1 May 2023)), which contains 1639 confirmed and 1126 predicted TFs. The intersection between the DEG list and the TF database identified 127 genes, forming the WB-TF dataset.

**Figure 2:**
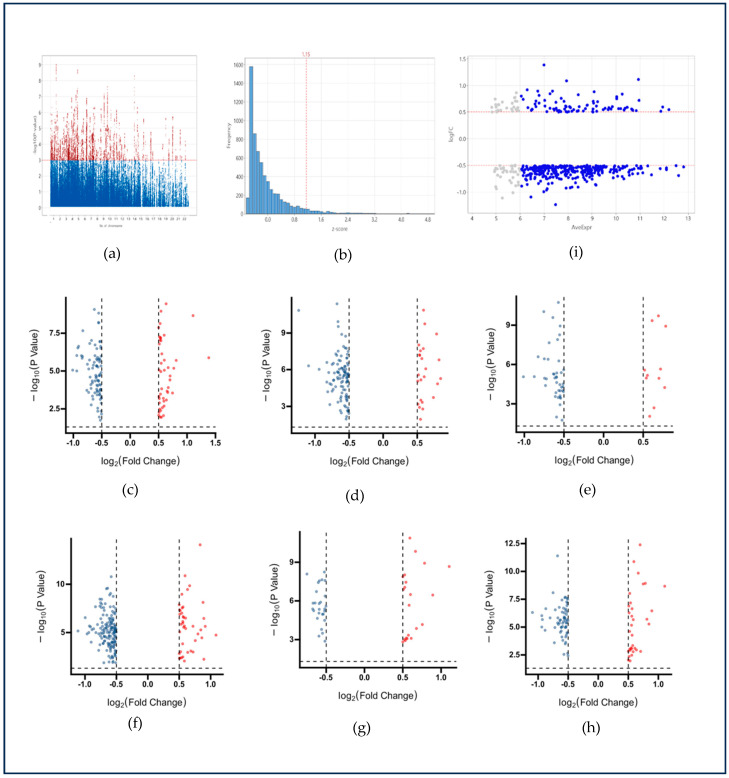
Identification and filtering of CRC-related circulating transcripts from publicly available datasets. (**a**) Manhattan plot of GWAS dataset showing 15,281 CRC-associated SNPs (*p* < 0.001). (**b**) Z-score distribution from eQTL dataset; transcripts with Z > 1.15 were retained. (**c**–**h**) Volcano plots showing differentially expressed transcripts (red: upregulated; blue: downregulated) in CRC across six datasets: (**c**) WB-Hub, (**d**) WB-TF, (**e**) WB-GWAS, (**f**) WB-eQTL, (**g**) WB-DigSeE, and (**h**) WB-CoReCG. (**i**) MA plot of WB-PADs dataset; transcripts with average expression > 6 were retained as biomarker candidates. Grey dots represent transcripts with low average expression (average expression < 6).

To incorporate genetic susceptibility evidence, the GWAS Catalog was queried using the term “colorectal cancer,” yielding 124 summary-level entries encompassing 3793 CRC patients and 410,350 healthy individuals. After harmonization, 15,281 SNPs corresponding to 1592 genes were retrieved. Intersection with the DEG list identified 53 overlapping transcripts, designated as the WB-GWAS dataset (Figure 2a).

Gene regulation at the transcriptional level was also evaluated using cis-expression quantitative trait loci (cis-eQTL) data from the eQTLGen Consortium (https://www.eqtlgen.org (accessed on 1 April 2023)), comprising association data for 31,684 genes in whole blood. Genes with Bonferroni-adjusted *p*-values < 0.05 and |Z| > 1.15 were retained (1289 genes). Intersection with the 956 DEGs yielded 194 overlapping transcripts, constituting the WB-eQTL dataset (Figure 2b).

To confirm disease-specific associations, two curated CRC gene databases were additionally examined. The DigSeE database identified 8866 genes linked to “colorectal cancer” through literature text mining, of which 2350 mutation-associated genes were selected. Intersection analysis revealed 48 overlapping DEGs, defined as the WB-DigSeE dataset. The CoReCG database, which includes 2056 experimentally validated CRC-associated genes from 2486 publications, was also intersected with the DEGs, resulting in 97 overlapping genes designated as the WB-CoReCG dataset.

Collectively, these analyses generated six evidence-based subsets—WB-Hub, WB-TF, WB-GWAS, WB-eQTL, WB-DigSeE, and WB-CoReCG (Table 1). Integration of these complementary datasets yielded a total of 440 distinct transcripts, representing a refined and biologically supported set of CRC-related genes. These were subsequently consolidated into the WB-PADs dataset for further expression-based filtering and cross-validation, referred to as the publicly available dataset (PAD) candidate grouping (Table 1).

An additional filtering step was then applied to ensure consistent detectability across clinical samples. Based on average expression levels visualized in the MA plot, transcripts with low abundance (mean expression < 6) were excluded. This threshold minimized the risk of technical dropout in qPCR validation and improved reliability for clinical application. After this filtering, 375 high-confidence transcripts remained, collectively designated as the WB-PADs dataset, which was subsequently used for cross-comparison and clinical validation studies (Figure 2c–i). Functional enrichment analysis of the 375 PAD genes revealed significant involvement in biological processes associated with tumor–immune interactions. GO biological process terms were highly enriched in cilia assembly, small GTPase-mediated signal transduction, and metabolic remodeling, indicating potential dysregulation of epithelial integrity and intracellular communication in CRC. GO molecular function categories highlighted phosphatase and O-methyltransferase activity, reflecting altered post-translational and metabolic regulatory mechanisms. GO cellular component enrichment suggested localization to post-synaptic regions, clathrin-coated vesicles, and Golgi-associated structures, supporting disruption of vesicle transport and signaling machinery during CRC progression. KEGG pathway analysis further demonstrated enrichment in cell adhesion molecules (CAMs), hematopoietic cell lineage, cellular senescence, and platinum-drug resistance, collectively indicating that these PAD genes capture both immune remodeling and therapeutic response-related transcriptional alterations in whole blood from CRC patients (Figure S2).

### 2.3. Construction of the WB-K Dataset

To determine whether the CRC-associated transcripts identified from public datasets were reproducible across different populations and sequencing platforms, independent RNA sequencing was performed using whole-blood samples collected from a Korean clinical cohort (10 CRC patients and 10 healthy controls, Table 2). This analysis aimed to verify that candidate biomarkers were not confined to a specific study population or experimental batch, thereby supporting the identification of biologically robust and population-independent markers.

**Figure 3:**
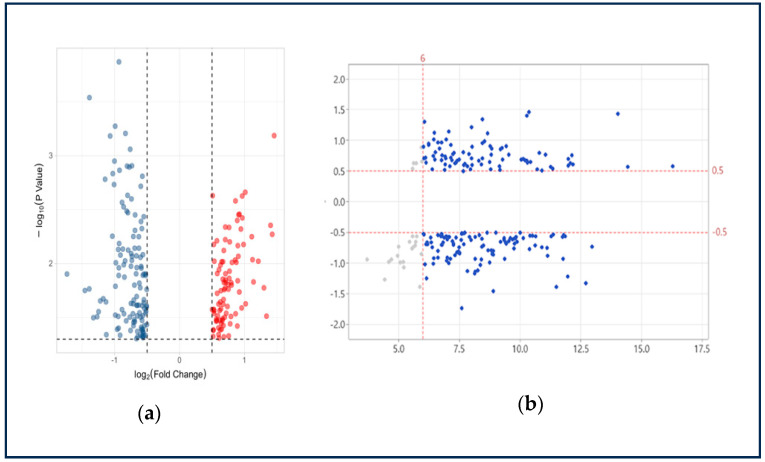
Transcript selection from the WB-K dataset. (**a**) Volcano plot showing differentially expressed transcripts (|log_2_FC| > 0.5, *p* < 0.05) in CRC vs. HC samples from the WB-K dataset (red: upregulated; blue: downregulated). (**b**) MA plot of differentially expressed genes in the discovery whole-blood RNA-seq cohort. Genes that met the predefined detectability and effect-size thresholds (mean expression > 6 and |log_2_FC| > 0.5) are shown in blue, indicating candidate transcripts suitable for clinical validation. Transcripts below these thresholds are shown in light gray. Red dashed lines indicate the cutoff values applied for detectability (vertical line at mean expression = 6) and minimum effect size (horizontal lines at log_2_FC = ±0.5).

Differential gene expression analysis was performed using criteria consistent with those applied to the WB-PADs dataset (|log_2_FC| > 0.5, *p* < 0.05, and mean expression > 6) to ensure methodological comparability and facilitate cross-validation between datasets. Using these thresholds, a total of 217 differentially expressed transcripts were identified and designated as the WB-K dataset (Figure 3). These transcripts represent CRC-associated expression changes observed in an independent Korean cohort and were subsequently used for integrative comparison with the WB-PADs dataset to identify reproducible, high-confidence circulating RNA biomarkers.

### 2.4. Selection of Candidate Genes for Clinical Validation

To identify reproducible circulating transcripts consistently associated with CRC across independent datasets, an intersection analysis was performed between the WB-PADs and WB-K datasets. This cross-comparison aimed to pinpoint genes that demonstrated concordant differential expression patterns in both publicly available and Korean cohort-derived data, thereby increasing biological robustness and reducing dataset-specific bias.

**Figure 4:**
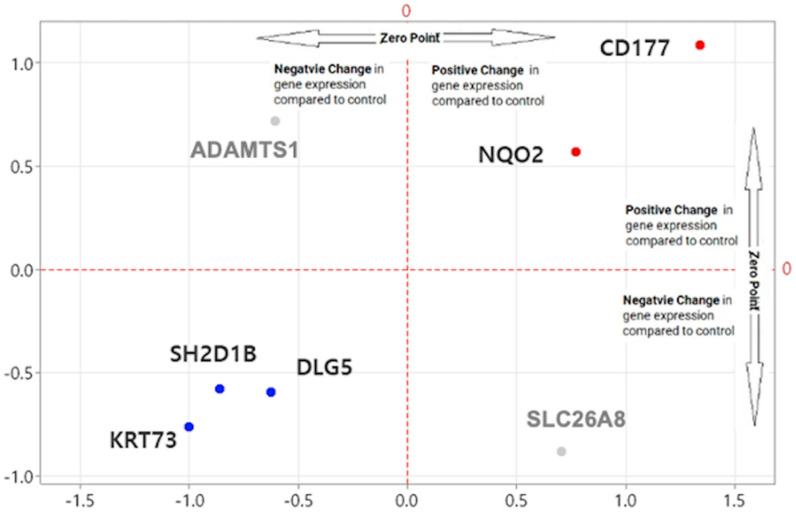
Scatter plot comparing fold changes of overlapping genes between the WB-PADs and WB-K datasets. Each point represents one gene, with the *x*- and *y*-axes indicating log_2_ fold changes relative to healthy controls in the WB-PADs and WB-K datasets, respectively. The intersecting dashed lines at (0, 0) denote the reference threshold where no expression change is observed. Genes plotted in blue exhibit consistently regulated patterns across datasets (either up- or down-regulated), reinforcing their reproducibility for subsequent validation. In contrast, *ADAMTS1* and *SLC26A8* (gray dots) demonstrate discordant trends.

The intersection yielded seven overlapping genes: *ADAMTS1*, *DLG5*, *CD177*, *SH2D1B*, *NQO2*, *KRT73*, and *SLC26A8*. The expression fold changes in these genes were visualized in a scatter plot, where the *x*-axis represented fold change in the WB-K dataset and the *y*-axis represented fold change in the WB-PADs dataset (Figure 4). The intersection at (0, 0) denoted no expression change relative to controls.

Among the seven overlapping genes, *CD177* and *NQO2* showed consistent upregulation, whereas *DLG5*, *SH2D1B*, and *KRT73* were consistently downregulated across both datasets. In contrast, *ADAMTS1* and *SLC26A8* exhibited discordant expression patterns between datasets and were therefore excluded from subsequent validation.

Consequently, five genes—*DLG5*, *CD177*, *SH2D1B*, *NQO2*, and *KRT73*—were selected as robust and reproducible candidates for clinical validation (Table 3). Notably, *CD177* encodes a neutrophil surface glycoprotein involved in innate immune activation, while *NQO2* is associated with redox homeostasis, both of which are crucial in the inflammatory tumor microenvironment. *DLG5* and *KRT73* are structural and epithelial-associated genes potentially linked to epithelial–mesenchymal transition (EMT), and *SH2D1B* encodes an immune adaptor protein that modulates lymphocyte signaling. The biological relevance of these genes supports their potential utility as non-invasive circulating biomarkers for CRC potential diagnostic relevance.

### 2.5. Clinical Validation of Five Circulating Transcripts Using RT-qPCR

To confirm whether the candidate transcripts identified through integrative transcriptomic analysis were reproducibly detectable and clinically relevant in a larger independent cohort, reverse transcription quantitative polymerase chain reaction (RT-qPCR) was performed. This validation aimed to verify that the selected transcripts (*DLG5*, *CD177*, *SH2D1B*, *NQO2*, and *KRT73*) could serve as robust circulating biomarkers capable of distinguishing CRC patients from healthy individuals in a real-world clinical setting.

**Figure 5:**
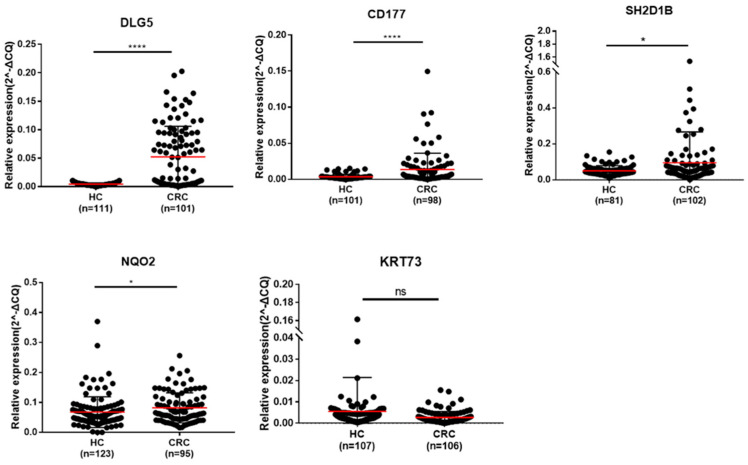
Expression levels of five candidate transcripts in healthy controls and colorectal cancer (CRC) patients. Scatter plots depict the relative expression of *DLG5*, *CD177*, *SH2D1B*, *NQO2*, and *KRT73* as measured by RT-qPCR in whole-blood samples from healthy controls and CRC patients. Red bars indicate the median and interquartile range. Four transcripts (*DLG5*, *CD177*, *SH2D1B*, and *NQO2*) showed significantly increased expression in CRC compared to HCs, while *KRT73* exhibited a non-significant downward trend. Statistical significance was determined using unpaired *t*-tests. *p* < 0.05 (*), *p* < 0.0001 (****) and ns = not significant.

Whole-blood RNA samples from 229 participants—including 106 CRC patients and 123 healthy controls (HCs)—were analyzed using the same normalization and quantification strategy applied in previous experiments (Figure 5). Among the five transcripts, *DLG5*, *CD177*, *SH2D1B*, and *NQO2* showed significantly increased expression in CRC samples compared with controls (unpaired *t*-test, *p* < 0.05). In contrast, *KRT73* exhibited a downward trend in expression among CRC patients, although this difference did not reach statistical significance (*p* > 0.05). A detailed summary of cross-dataset consistency in expression trends for these five transcripts is provided in Supplementary Table S3.

These findings provide independent clinical evidence supporting the diagnostic potential of the four upregulated transcripts—*DLG5*, *CD177*, *SH2D1B*, and *NQO2*—as blood-based RNA biomarkers for CRC detection. The overall expression trends were consistent with those observed in the discovery datasets, reinforcing their reproducibility and translational applicability for non-invasive CRC diagnosis.

### 2.6. Diagnostic Model Establishment and Performance Evaluation

**Figure 6:**
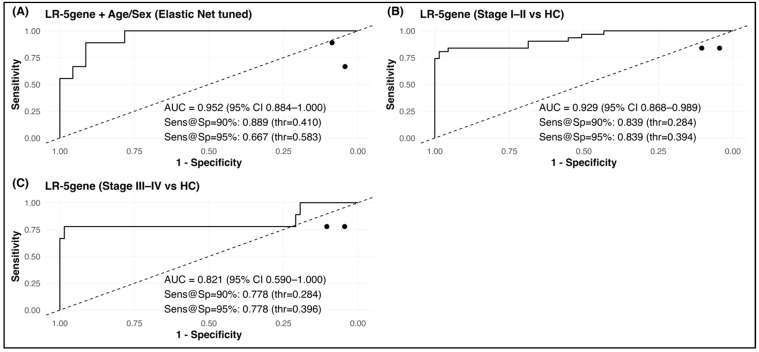
Diagnostic performance of the LR-5gene classifier in the RT-qPCR cohort. (**A**) Overall CRC vs. control discrimination with Elastic Net-tuned logistic regression. (**B**) Early-stage (Stage I–II) CRC vs. control. (**C**) Late-stage (Stage III–IV) CRC vs. control. Dashed line: random classifier. AUC values include 95% CI. Sensitivity values correspond to specificity = 90% and 95%.

To determine the clinical diagnostic value of the five-gene signature, logistic regression-based classifiers were developed using RT-qPCR expression data from whole-blood samples. After adjusting for age and sex, the multivariable LR-5gene model demonstrated strong diagnostic accuracy, achieving an AUC of 0.952 (95% CI 0.884–1.000) with sensitivities of 0.889 and 0.667 at fixed specificities of 90% and 95%, respectively (Figure 6A). These findings highlight the robustness of the transcriptomic signature independent of demographic confounders. To further confirm that demographic variables did not introduce bias into model training, age and sex distributions were compared between the training and validation cohorts within each diagnostic group. As summarized in Supplementary Table S1, no significant differences were observed in age or sex composition, indicating that the clinical characteristics of the two cohorts were comparable and unlikely to affect the model’s generalizability.

Given the importance of early detection for improving patient prognosis, stage-stratified classification was further performed. The model maintained high performance in early-stage (Stage I–II) CRC, yielding an AUC of 0.929 (95% CI 0.868–0.989) (Figure 6B), supporting its suitability for population screening. Performance remained acceptable for advanced CRC (Stage III–IV) with an AUC of 0.821 (95% CI 0.590–1.000) (Figure 6C), confirming diagnostic utility across diverse disease stages.

Furthermore, to assess cross-cohort robustness, the five-gene logistic regression model was externally evaluated using the independent whole-blood RNA-seq dataset GSE164191. The classifier achieved an AUC of 0.797 (95% CI 0.718–0.877) with clinically relevant sensitivity at high-specificity thresholds, supporting the generalizability of the whole-blood RNA signature beyond the Korean cohort (Figure S3).

Collectively, these results demonstrate that the five-gene panel offers high diagnostic accuracy in real-world clinical blood samples and shows particular promise for minimally invasive early CRC detection. Additionally, comparative ROC performance metrics for both individual genes and the multigene model are summarized in Supplementary Table S2 to further illustrate the additive predictive value of the combined signature. Although our model achieved high AUC values in both discovery and validation cohorts, these results should be interpreted cautiously. The 5-gene panel remains in the pre-clinical discovery stage, and head-to-head evaluation against FIT or other established diagnostic tools, as well as validation across diverse ethnic populations, will be essential future steps prior to clinical translation.

## 3. Discussion

While colonoscopy remains the diagnostic gold standard for colorectal cancer (CRC), its invasiveness and the resulting low compliance highlight the need for complementary, non-invasive approaches. Stool-based assays such as the fecal immunochemical test (FIT) and multitarget DNA tests offer alternatives, yet their sensitivity for adenomas and early-stage CRC remains limited. Liquid biopsy has therefore emerged as a promising tool for cancer diagnostics, with most CRC studies focusing on circulating tumor DNA (ctDNA) or circulating tumor cells (CTCs) [1,9]. However, these markers primarily capture tumor-derived fragments released at later disease stages, limiting their effectiveness for early detection [10,11].

In contrast, blood-based RNA profiling enables the detection of dynamic transcriptional changes arising not only from tumor cells but also from immune and stromal components, thereby reflecting early systemic responses to tumor development [3,9].

In this study, a reproducible five-gene signature (*DLG5*, *CD177*, *SH2D1B*, *NQO2*, and *KRT73*) was identified from whole-blood RNA that distinguished CRC patients from healthy controls. By integrating multiple GEO datasets with an independent Korean RNA-seq cohort and validating results through RT-qPCR in clinical samples, we established a multi-layered discovery framework that enhanced both biological relevance and reproducibility. The integration of functional annotation resources—such as transcription factor, eQTL, GWAS, and disease-gene databases—further minimized false positives and ensured that the selected genes were mechanistically linked to CRC pathogenesis.

A distinguishing aspect of this study is the use of whole blood rather than plasma or peripheral blood mononuclear cells (PBMCs). Previous transcriptomic studies have primarily analyzed PBMCs, which contain lymphocytes, monocytes, NK cells, and dendritic cells but exclude granulocytes and other myeloid-derived populations [5]. These excluded cell types, particularly neutrophils and macrophage precursors, play central roles in tumor-associated inflammation [4]. Whole-blood RNA encompasses the full leukocyte spectrum as well as platelets and erythrocyte-derived transcripts [12], providing a more comprehensive representation of the systemic immune landscape. This broader coverage likely explains why immune-related genes such as *CD177*, *SH2D1B*, and *NQO2* were identified here but have been underrepresented in PBMC-based or cfRNA studies. Unlike PBMCs, whole-blood RNA encompasses transcripts derived from granulocytes, platelets, and erythroid precursors, which participate in systemic immune and metabolic reprogramming during tumor development. Therefore, analyzing total blood RNA allows for a more comprehensive view of host–tumor crosstalk. Thus, our findings underscore the methodological advantage of whole-blood transcriptomics in capturing the complex interplay between tumor and host immunity in CRC.

Among the identified transcripts, *CD177*, *NQO2*, and *KRT73* displayed consistent expression across discovery and validation platforms. *CD177*, a neutrophil activation marker, was strongly upregulated, consistent with elevated neutrophil-to-lymphocyte ratios and proinflammatory signatures reported in CRC [12,13,14]. *NQO2*, involved in cellular redox regulation, was similarly upregulated, suggesting a compensatory response to oxidative stress in tumor-bearing individuals [15,16]. Conversely, *KRT73*, associated with epithelial differentiation, showed a downregulation trend, potentially reflecting epithelial–mesenchymal transition (EMT) and loss of epithelial polarity during tumor progression [17,18].

Notably, *DLG5* and *SH2D1B* exhibited opposite expression trends between RNA-seq discovery and RT-qPCR validation, suggesting context-dependent or cell-specific regulation. *DLG5*, a scaffold protein essential for epithelial integrity, may be suppressed in tumor tissues but upregulated in circulation through inflammation-induced signaling pathways such as NF-κB and MAPK activation [19,20]. *SH2D1B*, encoding the adaptor protein EAT-2 that modulates SLAM-family signaling in NK and T cells, could be dynamically expressed depending on immune activation status. Alternatively, these discrepancies may reflect technical factors such as primer specificity, isoform coverage, or differences in the cellular composition of bulk versus targeted assays [21]. To acknowledge potential interpretation differences across transcriptomic platforms, we note that gene expression values were derived from log_2_(CPM)-scaled RNA-seq datasets and log_2_-normalized probe intensities in microarray datasets. Although a common mean expression >6 threshold was applied to ensure strong biological detectability and reliable downstream qPCR validation, we recognize that technical scale discrepancies could contribute to minor variations in fold-change magnitude and direction across cohorts. Therefore, the biological convergence of expression patterns across datasets, rather than exact numeric equivalence, informed the prioritization of clinically measurable biomarkers in this study. Together, these findings suggest that whole-blood RNA integrates signals from multiple cellular sources, reflecting both steady-state and reactive transcriptional programs within the host–tumor interface. Given the modest sample size of the WB-K cohort, the nominal *p*-value thresholds applied in this dataset may increase the risk of false-positive detection. However, as WB-K was not relied upon as a standalone discovery source and candidate genes were selected only when reproducible across independent datasets, the risk was mitigated. Even so, this statistical limitation should be validated in future studies with larger external discovery cohorts.

Although *DLG5* and *SH2D1B* were downregulated in both public RNA-seq cohorts, their expression appeared upregulated in our clinical qPCR validation. This discrepancy may arise from several biological and technical factors. First, whole-blood RNA expression is strongly influenced by cellular composition, particularly the relative abundance of circulating immune subpopulations. Changes in neutrophil or NK-cell proportions between datasets could alter the predominant cellular source of each transcript, thereby affecting its direction of change when measured at the bulk-blood level. Second, platform-specific detection differences—including probe/primer target region selection, isoform coverage, and 3′ end quantification biases in qPCR—may capture different transcript variants or post-transcriptional regulation effects. Third, CRC patients recruited at different disease states or physiological conditions (e.g., inflammatory responses associated with tumor progression) may also contribute to heterogeneous expression patterns.

Despite this direction inconsistency, both *DLG5* and *SH2D1B* demonstrated statistically significant discriminatory power when assessed individually and provided essential complementary information that improved the performance of the multigene logistic model. Importantly, the three genes with full directional reproducibility across cohorts (*CD177*, *NQO2*, and *KRT73*) constitute the most stable core of the panel, while *DLG5* and *SH2D1B* enhance model sensitivity and classification efficiency when integrated in a combined signature. Future work using cell-type-resolved transcriptomics and isoform-specific assays will be necessary to further clarify the mechanistic underpinnings of these gene-level differences.

To further clarify the cellular origins of these biomarkers, we referenced established whole-blood expression atlases. *CD177* and *FCGR1A* strongly point toward neutrophils, monocytes, and T/B-lymphocyte involvement, highlighting the dominant contribution of circulating immune effector populations. Meanwhile, *DLG5* and *SH2D1B*, despite showing discordant directionality between discovery and validation datasets, map to epithelial–immune interaction pathways and NK/T-cell signaling, respectively. Therefore, these discrepancies likely reflect context-dependent immune activation rather than technical artifacts.

To align this interpretation with diagnostic performance, we emphasize that *CD177*, *NQO2*, and *KRT73* demonstrated the most stable cross-platform consistency, while *DLG5* and *SH2D1B* were retained due to their additive contribution to multigene classifier performance.

From a clinical benchmarking perspective, unlike plasma-based SEPT9 methylation assays—which primarily detect tumor-derived DNA shedding at later disease stages—our whole-blood RNA signature captures early immunometabolic disturbance, enabling enhanced discrimination of Stage I–II CRC. This advantage directly addresses a critical unmet need in population-based CRC screening.

Collectively, the five-gene panel identified in this study reflects both tumor-intrinsic and immune-mediated alterations, suggesting its potential application in early CRC potential diagnostic relevance. The use of whole blood as a diagnostic matrix also offers practical and biological advantages: it requires minimal preprocessing, preserves RNA integrity across all circulating cell types, and captures early immunometabolic shifts associated with tumorigenesis. While whole-blood transcriptome profiling enables practical and minimally invasive detection, the cellular sources of circulating RNA remain diverse, including leukocytes, platelets, and erythrocyte-derived vesicles. Therefore, the transcriptional changes observed in this study cannot be assigned to a specific immune-cell population at this stage. Future investigation incorporating cell-type deconvolution or sorted-cell RNA-seq will be required to elucidate the mechanistic origin of these biomarkers. Moreover, the integrative analytical approach—combining public transcriptome resources, functional annotation, and clinical validation—demonstrates a scalable model for developing population-independent, blood-based RNA diagnostics.

From a translational perspective, incorporating such transcriptomic signatures into diagnostic models may enhance clinical utility. In future work, we plan to apply supervised machine-learning algorithms—such as logistic regression, random forest, and gradient boosting—to optimize feature selection and construct predictive panels supported by multi-layer biological evidence. This strategy will enable quantitative weighting of molecular predictors and improve diagnostic performance, stage discrimination, and reproducibility across diverse populations. Furthermore, integrating longitudinal samples and treatment-response data may clarify whether the five-gene pathway provides value for real-time monitoring and early detection in high-risk cohorts.

In summary, whole-blood RNA profiling provides a holistic view of systemic transcriptional alterations in CRC, bridging tumor biology and host immune responses. The identified five-gene signature—representing epithelial, immune, and metabolic processes—illustrates the potential of whole-blood transcriptomics as a minimally invasive, clinically actionable platform for CRC detection and disease monitoring.

## 4. Materials and Methods

### 4.1. Public Dataset Selection and Processing

Two independent whole-blood transcriptome datasets were retrieved from the Gene Expression Omnibus (GEO) database by searching with the keywords “colorectal cancer” and “blood.” Datasets were included if they met the following criteria: (1) transcriptome data derived from whole-blood samples, and (2) availability of untreated CRC samples and healthy controls for comparison. Based on these criteria, two datasets were selected: (1) GSE164191 (RNA sequencing, Illumina HiSeq 2500, San Diego, CA, USA), comprising 59 CRC and 62 normal blood samples, and (2) GSE11545 (microarray, Affymetrix Human Genome U133 Plus 2.0 Array, Thermo Fisher Scientific, Waltham, MA, USA), from which 9 CRC and 9 control samples were selected for analysis.

Because these datasets were generated on different platforms (RNA-seq and microarray), direct comparison required normalization and batch-effect correction. After probe-level filtering, low-variance and low-expression features were excluded, and expression values were mapped to Entrez gene IDs. Batch effects were subsequently corrected using the ComBat method, which applies an empirical Bayesian framework to harmonize expression profiles across studies. Following this preprocessing, the two datasets were integrated to generate a unified whole-blood (WB) dataset for downstream differential expression analysis. To visually assess the effectiveness of batch correction, principal component analysis (PCA) was conducted before and after applying ComBat. As shown in Supplementary Figure S1, CRC and control samples remained biologically separable after correction, while the dataset-specific bias observed prior to ComBat was markedly reduced. These results confirm that batch harmonization successfully improved cross-platform comparability.

The integrated WB dataset was used to perform differential expression analysis between CRC and normal controls. A total of 956 differentially expressed genes (DEGs) were identified, which were considered CRC-associated transcripts for subsequent analyses.

### 4.2. PAD Filtering and Cross-Dataset Validation

DEGs identified from the integrated WB dataset (FDR < 0.05, |log_2_FC| > 0.5) were evaluated against multiple CRC-related resources to prioritize biologically supported candidates. DEGs were projected onto the STRING PPI network, and hub genes were defined as nodes with degree >10. Transcription factor-associated genes were obtained from the Human TFs database. CRC-associated variants were collected from the GWAS Catalog (significance threshold: *p* < 0.001) and mapped to genes; whole-blood cis-eQTLs were retrieved from the eQTLGen Consortium (Bonferroni-adjusted *p* < 0.05, |Z| > 1.15). Disease-gene associations were cross-checked using DigSeE and CoReCG. Genes supported by at least one of these resources were retained; additionally, we required mean expression ≥6 (MA-plot) to ensure detectability in clinical specimens. The resulting set was termed WB-PADs. Candidates consistently differentially expressed in both WB-PADs and the Korean RNA-seq cohort (WB-K) were considered for RT-qPCR validation. A detectability filter of mean expression >6 was applied consistently across platforms to ensure reliable transcript measurement in blood. For RNA-seq datasets, expression values refer to log_2_(CPM) after TMM normalization, whereas microarray datasets reflect log_2_-normalized probe intensities.

### 4.3. Generation of the WB-K Dataset Through Whole-Blood RNA Sequencing and Differential Expression Analysis

Whole-blood RNA sequencing (RNA-seq) was performed to identify novel CRC-associated transcripts specific to the Korean population. Total RNA was extracted from 10 colorectal cancer (CRC) and 10 healthy control (HC) samples collected in Tempus™ Blood RNA Tubes (Thermo Fisher Scientific, Waltham, MA, USA). RNA concentration was measured using Quant-iT RiboGreen (Invitrogen, Carlsbad, CA, USA), and RNA integrity was assessed using the Agilent 2100 Bioanalyzer (Agilent Technologies, Santa Clara, CA, USA). Only samples with RNA integrity number (RIN) ≥7.0 were used for library preparation.

RNA sequencing and differential expression analysis were conducted by Macrogen Inc. (Seoul, Republic of Korea). RNA libraries were prepared using TruSeq Stranded Total RNA with Ribo-Zero Globin Kit (Illumina, San Diego, CA, USA) following the manufacturer’s protocol. Briefly, rRNA was depleted from total RNA, and the remaining RNA was fragmented and reverse-transcribed into first-strand cDNA using SuperScript II reverse transcriptase (Invitrogen) and random primers. Second-strand synthesis was performed using DNA Polymerase I, RNase H, and dUTP, followed by end repair, 3′ adenylation, adapter ligation, and PCR amplification to generate the final cDNA library. Libraries were quantified using the KAPA Library Quantification Kit (KAPA Biosystems, Basel, Switzerland) and evaluated with the D1000 ScreenTape System (Agilent Technologies).

Sequencing was performed on an Illumina NovaSeq 6000 platform to generate 150-bp paired-end reads. Raw reads underwent quality control using FastQC, adapter trimming with Trimmomatic, and alignment to the human reference genome (GRCh38) using HISAT2 (version 2.1.0). Transcript assembly was performed using StringTie (version 2.1.3b) to reconstruct transcript models and estimate expression levels. Gene-level counts were obtained using featureCounts, and normalization and differential expression analysis were conducted with DESeq2 in R (version 4.4.2).

Differentially expressed genes (DEGs) between CRC and HC groups were defined by the criteria |log_2_ fold change| > 0.5, *p* < 0.05, and mean normalized expression >6. Based on these thresholds, 217 transcripts were identified and designated as the WB-K dataset for downstream integration and validation analyses.

### 4.4. Cross-Dataset Integration and Visualization

Overlap between WB-PADs and WB-K datasets was identified using the dplyr (version 1.1.4) and VennDiagram packages (version 1.7.3) in R(version 4.4.2). Scatter and correlation plots comparing fold changes were generated using ggplot2 (version 3.5.1). Volcano and MA plots were produced using EnhancedVolcano (version 1.20.0) to visualize differential expression patterns and average transcript abundance.

### 4.5. Patient Cohorts

CRC samples were obtained from the Department of Gastroenterology, Severance Hospital, Seoul, Republic of Korea (IRB No. 4-2017-0148). HC samples were collected during routine health screening at Wonju Severance Christian Hospital (IRB No. CR319115). The inclusion criteria for patients included histological confirmation of CRC based on colonoscopy and histological results with dysplasia grade level, villous component protein, and size and number of polyps, according to the European Society of Gastrointestinal Endoscopy (ESGE). The exclusion criteria included prior CRC resection or evidence of hereditary colorectal cancer syndrome. The staging criteria for patients with CRC from stages I to IV followed the guidelines set forth by the ESGE. This classification was based on the widely accepted TNM staging system, ensuring consistency with the ESGE standards for accurate patient stratification and treatment planning. The HC group included individuals with no significant findings after colonoscopy and no other cancers. Informed consent was obtained from all the participants. The clinicopathologic characteristics of participants are summarized in Table 4.

### 4.6. Clinical Samples and RNA Extraction

Blood samples were collected by venipuncture into 3 mL Tempus™ Blood RNA Tubes as a secondary or subsequent draw to avoid epithelial contamination. Tubes were vortexed for 10 s to ensure complete mixing with 6 mL stabilizing reagent and stored upright at room temperature (18–25 °C) for up to 5 days before processing, or refrigerated (2–8 °C) or frozen (−20 °C) as needed.

RNA was extracted using a Tempus Spin RNA Isolation Kit (Thermo Fisher Scientific) following the manufacturer’s protocol. The quality of the isolated RNA was assessed using an RNA 6000 Nano LabChip with an Agilent 2100 Bioanalyzer (Agilent Technologies, Santa Clara, CA, USA) and a NanoDrop spectrophotometer (Thermo Fisher Scientific).

Complementary DNA (cDNA) was synthesized using a High-Capacity cDNA Reverse Transcription Kit (Thermo Fisher Scientific). RNA calculated based on the measured concentration was diluted with nuclease-free water to achieve a concentration of 2 μg/14.2 μL. A mixture was prepared using 10× RT buffer, 25× deoxynucleotide triphosphate (dNTP) mix, 10× RT random primers, and MultiScribe^TM^ Reverse Transcriptase (Invitrogen, Carlsbad, CA, USA) at a ratio of 10:4:10:5. All reagents were contained in a High-Capacity cDNA Reverse Transcription Kit (Applied Biosystems, Foster City, CA, USA). The total volume of the reaction mixture was modified according to the number of samples, and 5.8 μL of the mixture was distributed into each sample. Each sample was mixed thoroughly and briefly centrifuged to ensure even distribution.

Subsequently, cDNA was synthesized using random hexamers and dNTPs in a thermal cycler (Bio-Rad Laboratories, Hercules, CA, USA). The reaction was initiated at 25 °C for 10 min to allow priming and enzyme activation (Step 1), followed by incubation at 37 °C for 50 min to facilitate reverse transcription (Step 2). Subsequently, the reaction mixture was heated to 85 °C for 5 min to inactivate the reverse transcriptase enzyme (Step 3). All steps were conducted in accordance with the manufacturer’s instructions.

### 4.7. Reverse Transcription-Quantitative PCR

Expression levels of candidate genes were quantified using TaqMan^®^ Array Custom Cards on a QuantStudio™ 7 Pro Real-Time PCR System (Thermo Fisher Scientific). Each reaction contained 50 µL of TaqMan^®^ Fast Advanced Master Mix and 50 µL of cDNA template, run in duplicate per sample. 

GAPDH served as the internal control. Relative expression was calculated using the ΔΔCt method:

$$\Delta \Delta Ct=[Ct_{\mathrm{target},\mathrm{test}}-Ct_{\mathrm{ref},\mathrm{test}}]-\left[Ct_{\mathrm{target},\mathrm{calibrator}}-Ct_{\mathrm{ref},\mathrm{calibrator}}\right]$$

Expression fold-changes were expressed as 2^−ΔΔCt^.

### 4.8. Protein–Protein Interaction (PPI) Network Analysis

Differentially expressed genes (DEGs) were imported into the STRING database (version 12.0; https://string-db.org (accessed on 9 September 2025)) to construct a protein–protein interaction (PPI) network with a confidence score >0.7. The network was visualized using Cytoscape (version 3.8.2) with the StringApp plugin (version 1.6.0). Topological parameters were analyzed using the NetworkAnalyzer tool, and hub genes were defined as nodes with a connectivity degree >10. These hub genes were designated as the WB-Hub dataset for subsequent integration with other evidence layers.

### 4.9. Logistic Regression Modeling and External Validation

A multivariable logistic regression classifier was constructed using the standardized expression of the five selected transcripts (*DLG5*, *CD177*, *SH2D1B*, *NQO2*, and *KRT73*). The model was trained in the RT-qPCR cohort with CRC status as the response variable. Model performance was evaluated based on ROC curves, AUC with 95% CIs (DeLong), and sensitivity at fixed specificities of 90% and 95%.

For external validation, the trained model was independently applied to the whole-blood RNA-seq dataset GSE164191. Expression matrices and clinical metadata were preprocessed to ensure consistent sample matching, and the same z-score transformation and linear predictor were used to generate predicted CRC probabilities for ROC analysis.

### 4.10. Statistical Analysis

Two-group comparisons were performed using unpaired Student’s *t*-tests in GraphPad Prism (version 9.0; GraphPad Software, San Diego, CA, USA). Statistical significance was set at *p* < 0.05. All plots were generated using ggplot2 in R (version 4.4.2, R Foundation for Statistical Computing, Vienna, Austria).

## 5. Conclusions

This study identified and validated a reproducible five-gene panel—*DLG5*, *CD177*, *SH2D1B*, *NQO2*, and *KRT73*—derived from whole-blood RNA, with strong diagnostic relevance for colorectal cancer (CRC). Through the integration of public transcriptome datasets, genomic annotation resources, and independent Korean clinical cohorts, a robust multi-layered framework was established for discovering and validating non-invasive CRC biomarkers.

The identified transcripts collectively represent epithelial, immune, and metabolic pathways, reflecting the dual contribution of tumor biology and systemic host responses. Notably, the inclusion of neutrophil- and myeloid-associated genes such as *CD177*, *SH2D1B*, and *NQO2* underscores the capacity of whole-blood transcriptomics to capture inflammation-driven molecular alterations that are often missed in plasma or PBMC-based assays. This highlights the methodological advantage of whole-blood RNA profiling as a comprehensive platform for monitoring tumor–host interactions.

Compared with traditional invasive procedures such as colonoscopy or the limited sensitivity of stool-based tests, whole-blood RNA analysis provides a minimally invasive, scalable approach that can enhance early CRC detection and patient compliance. When integrated with advanced analytic tools such as machine learning-based algorithms, these transcriptomic signatures hold promise for improving diagnostic accuracy, stage discrimination, and longitudinal disease monitoring.

Future studies involving larger, multi-center cohorts and single-cell transcriptomic analyses are warranted to elucidate the cellular origins and biological mechanisms underlying these markers. We also acknowledge that the current sample size, although clinically validated, remains limited and therefore larger prospective studies will be needed to confirm the diagnostic robustness and real-world generalizability of this five-gene panel. Collectively, the findings demonstrate that whole-blood transcriptomic profiling captures systemic molecular changes associated with CRC and represents a promising foundation for developing next-generation, RNA-based diagnostic tools for early detection and clinical management of colorectal cancer.

## Figures and Tables

**Table 1:** Summary of DEGs Derived from Six Public Datasets Used to Construct the WB-PADs dataset.

No.	Dataset	Up-Regulated Genes	Down-Regulated Genes	Total
1	WB-Hub	45	87	132
2	WB-TF	24	103	127
3	WB-GWAS	11	42	53
4	WB-eQTL	43	151	194
5	WB-DigSeE	21	27	48
6	WB-CoReCG	33	64	97

**Table 2:** Demographic and clinical characteristics of participants in the WB-K RNA-seq cohort.

Characteristics	Healthy Control, *n* (%)	Colorectal Cancer, *n* (%)
Age (SD)	54.0 (7.6)	46.8 (8.1)
Gender (%)		
Male	5 (50.0)	5 (50.0)
Female	5 (50.0)	5 (50.0)

**Table 3:** Five candidate genes showing consistent differential expression patterns in both WB-PADs and WB-K datasets.

Gene Symbol	log_2_FC		*p*-Value	
	WB Dataset	WB-K Dataset	WB Dataset	WB-K Dataset
*DLG5*	−0.59	−0.63	0.0005	0.007
*CD177*	1.09	1.34	0.00001	0.03
*SH2D1B*	−0.58	−0.86	0.000001	0.01
*NQO2*	0.57	0.77	0.000002	0.01
*KRT73*	−0.76	−1.00	0.0001	0.001

**Table 4:** Clinicopathologic characteristics of study participants.

Characteristics	Healthy Control, *n* (%)	Colorectal Cancer, *n* (%)
Age, y (SD)	63.2 (11.2)	48.9 (9.8)
Gender, *n* (%)		
Male	60 (59.4)	64 (57.7)
Female	41 (40.6)	47 (42.3)

## Data Availability

Data is contained within the article and Supplementary Materials. In addition, the RNA-seq dataset generated from the WB-K cohort will be deposited in the Gene Expression Omnibus (GEO) repository, and the accession number will be provided once available.

## Supplementary Materials

The following supporting information can be downloaded at: https://www.mdpi.com/article/10.3390/ijms262311625/s1.

## References

[1] Reinert T., Henriksen T.V., Christensen E., Sharma S., Salari R., Sethi H., Knudsen M., Nordentoft I., Wu H.-T., Tin A.S. (2019). Analysis of Circulating Tumour DNA to Monitor Disease Burden Following Colorectal Cancer Surgery. Nat. Med..

[2] Cohen J.D., Li L., Wang Y., Thoburn C., Afsari B., Danilova L., Douville C., Javed A.A., Wong F., Mattox A. (2018). Detection and Localization of Surgically Resectable Cancers with a Multi-Analyte Blood Test. Science.

[3] Best M.G., Sol N., Kooi I., Tannous J., Westerman B.A., Rustenburg F., Schellen P., Verschueren H., Post E., Koster J. (2015). RNA-Seq of Tumor-Educated Platelets Enables Blood-Based Pan-Cancer, Multiclass, and Molecular Pathway Cancer Diagnostics. Cancer Cell.

[4] Grivennikov S.I., Greten F.R., Karin M. (2010). Immunity, Inflammation, and Cancer. Cell.

[5] Subrahmanyam Y.V., Yamaga S., Prashar Y., Lee H.H., Hoe N.P., Kluger Y., Gerstein M., Goguen J.D., Newburger P.E., Weissman S.M. (2001). RNA Expression Patterns Change Dramatically in Human Neutrophils Exposed to Bacteria. Blood.

[6] Park S.Y., Han J., Kim T.H., Lim J., Lee H., Choi H., Kim J., Lee H. (2022). Blood Test for Breast Cancer Screening through the Detection of Tumor-Associated Circulating Transcripts. Int. J. Mol. Sci..

[7] Han J., Kim T.H., Park S.Y., Lim J., Kim J., Choi H., Lee H. (2023). Diagnostic Performance of Tumor-Associated Circulating Transcripts in Colorectal Cancer. Cancers.

[8] Kim L.A., Han J., Kim T.I., Park J.J., Lee J.M., Kim J.K., Park S., Lee H. (2025). Circulating RNA Markers Associated with Adenoma–Carcinoma Sequence in Colorectal Cancer. Int. J. Mol. Sci..

[9] Keller L., Pantel K. (2019). Unravelling Tumor Heterogeneity by Single-Cell Profiling of Circulating Tumor Cells. Nat. Rev. Cancer.

[10] Bettegowda C., Sausen M., Leary R.J., Kinde I., Wang Y., Agrawal N., Bartlett B.R., Wang H., Luber B., Alani R.M. (2014). Detection of Circulating Tumor DNA in Early- and Late-Stage Human Malignancies. Sci. Transl. Med..

[11] Wan J.C.M., Massie C., Garcia-Corbacho J., Mouliere F., Brenton J.D., Caldas C., Pacey S., Baird R., Rosenfeld N. (2017). Liquid Biopsies Come of Age: Towards Implementation of Circulating Tumour DNA. Nat. Rev. Cancer.

[12] Haemmerle M., Stone R.L., Menter D.G., Afshar-Kharghan V., Sood A.K. (2018). The Platelet Lifeline to Cancer: Challenges and Opportunities. Cancer Cell.

[13] Ke S., Lei Y., Guo Y., Xie F., Yu Y., Geng H., Zhong Y., Xu D., Liu X., Yu F. (2024). *CD177* Drives the Transendothelial Migration of Treg Cells Enriched in Human Colorectal Cancer. Clin. Transl. Immunol..

[14] Zhou J., Xu Q., Liu H., Miao J., Bian C., Wei Y., Wang W., Jiang S. (2024). Prognostic Value of Tumor-Associated CD177+ Neutrophils in Lung Adenocarcinoma. Oncol. Lett..

[15] Lee D.-Y., Song M.-Y., Kim E.-H. (2021). Role of Oxidative Stress and Nrf2/KEAP1 Signaling in Colorectal Cancer: Mechanisms and Therapeutic Perspectives with Phytochemicals. Antioxidants.

[16] Hirose Y., Sakata J., Kobayashi T., Miura K., Yuza K., Nakano M., Ichikawa H., Nagahashi M., Shimada Y., Kameyama H. (2020). NQO1 as a Marker of Chemosensitivity and Prognosis for Colorectal Liver Metastasis: A Cohort Study. Anticancer Res..

[17] Yang Y., Feng M., Bai L., Yang H., Zou H., Zheng L., Song X., Liu H., Zhang W., Xiong Y. (2021). Comprehensive Analysis of EMT-Related Genes and lncRNAs in the Prognosis, Immunity, and Drug Treatment of Colorectal Cancer. J. Transl. Med..

[18] Pastushenko I., Blanpain C. (2019). EMT Transition States during Tumor Progression and Metastasis. Trends Cell Biol..

[19] Shin D.H., Lee H., Park J.H., Lee S.H., Kang H.J., Kim S.Y., Kim J., Lee H. (2021). *DLG5* Regulates Epithelial Integrity and Epithelial–Mesenchymal Transition in Colorectal Cancer. Mol. Cancer Res..

[20] Liu Y., Guo R., Hao G., Zhu S., Wang L., Wu Y., Xu M., Zhao X. (2020). *DLG5* Suppresses NF-κB Signaling to Maintain Epithelial Homeostasis. J. Biol. Chem..

[21] Veillette A. (2022). SLAM-Family Receptors: Immune Regulators with or without SAP-Family Adaptors. Cold Spring Harb. Perspect. Biol..

