# Supplementary material for: Identification of Colorectal Cancer-Related RNA Markers from Whole Blood Using Integrated Bioinformatics Analysis
Source: Int J Mol Sci. 2025 Nov 30;26(23):11625. doi: 10.3390/ijms262311625 (PMC12692440; doi:10.3390/ijms262311625)
Supplement: Supplementary file 1 [file ijms-26-11625-s001.zip › ijms-3940926-supplementary.pdf]

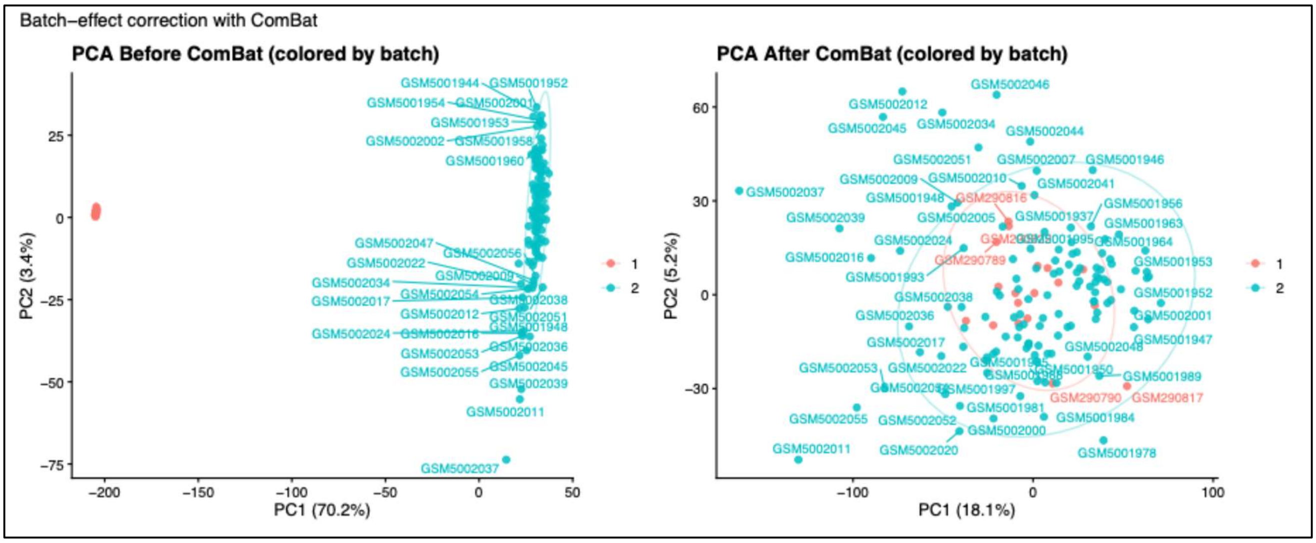

(A) (B)

Figure S1. Principal component analysis before and after ComBat batch correction. (A) PCA plot of the merged whole-blood transcriptome datasets before batch correction, colored by cohort (batch). Samples cluster primarily by dataset origin, indicating a strong batch effect. (B) PCA plot after ComBat-based batch-effect removal, showing improved mixing of samples from both cohorts along the principal components. After correction, CRC and healthy control samples are distributed without obvious batch-driven separation, supporting the suitability of the integrated expression matrix for downstream DEG and biomarker analyses.

gene_id

A

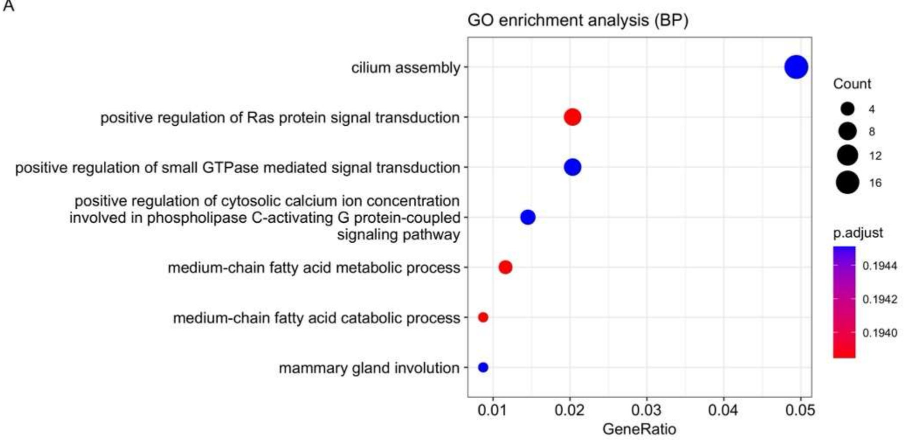
GO enrichment analysis (BP)
cilium assembly
positive regulation of Ras protein signal transduction
positive regulation of small GTPase mediated signal transduction
positive regulation of cytosolic calcium ion concentration involved in phospholipase C-activating G protein-coupled signaling pathway
medium-chain fatty acid metabolic process
medium-chain fatty acid catabolic process
mammary gland involution
0.01
0.02
0.03
0.04
0.05
GeneRatio
Count
4
8
12
16
p.adjust
0.1944
0.1942
0.1940

B

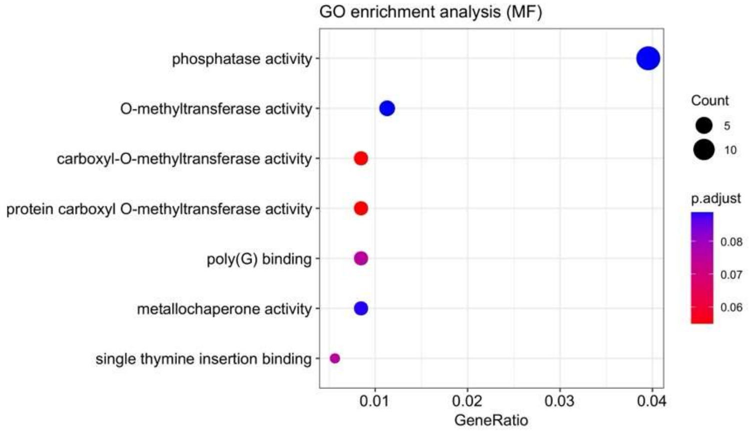
GO enrichment analysis (MF)
phosphatase activity
O-methyltransferase activity
carboxyl-O-methyltransferase activity
protein carboxyl O-methyltransferase activity
poly(G) binding
metallochaperone activity
single thymine insertion binding
0.01
0.02
0.03
0.04
GeneRatio
Count
5
10
p.adjust
0.08
0.07
0.06

C

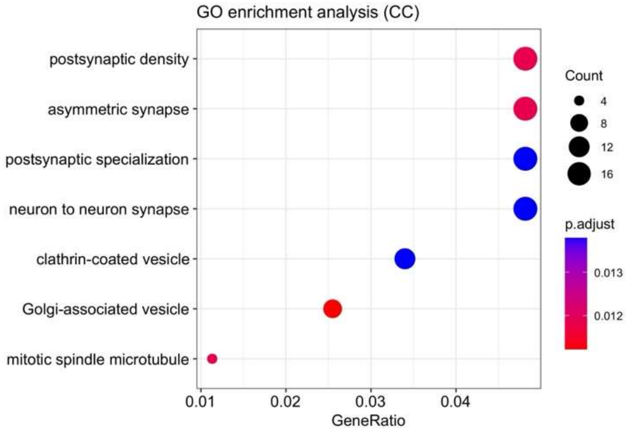
GO enrichment analysis (CC)
postsynaptic density
asymmetric synapse
postsynaptic specialization
neuron to neuron synapse
clathrin-coated vesicle
Golgi-associated vesicle
mitotic spindle microtubule
0.01
0.02
0.03
0.04
GeneRatio
Count
4
8
12
16
p.adjust
0.013
0.012

D

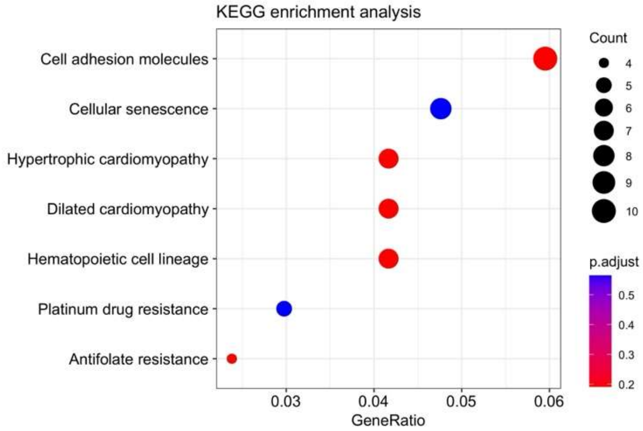
KEGG enrichment analysis
Cell adhesion molecules
Cellular senescence
Hypertrophic cardiomyopathy
Dilated cardiomyopathy
Hematopoietic cell lineage
Platinum drug resistance
Antifolate resistance
0.03
0.04
0.05
0.06
GeneRatio
Count
4
5
6
7
8
9
10
p.adjust
0.5
0.4
0.3
0.2

Figure S2. Functional enrichment analysis of the 375 PAD genes. (A) GO Biological Process (BP), (B) GO Molecular Function (MF), and (C) GO Cellular Component (CC) enrichment results, ranked by adjusted p-value. Top terms reflect involvement in immune activation, vesicle transport, and metabolic regulation in CRC. (D) KEGG pathway enrichment indicating associations with immune signaling (cell adhesion molecules, hematopoietic cell lineage), tumor-related senescence pathways, and chemotherapy response mechanisms.Dot size represents gene count per term; dot color indicates adjusted p-value.

Supplementary Table S1. Demographic characteristics of training and validation cohorts

Group	Cohort	Mean Age (yrs)	Sex (% Male / % Female)	Age p-value	Sex p-value
Healthy Controls (HC)	Training	49.3	65.9% / 34.1%	0.82	0.55
Healthy Controls (HC)	Test	48.3	47.8% / 52.2%		
CRC Patients	Training	63.6	41.9% / 58.1%	0.74	0.68
CRC Patients	Test	62.0	55.6% / 44.4%		

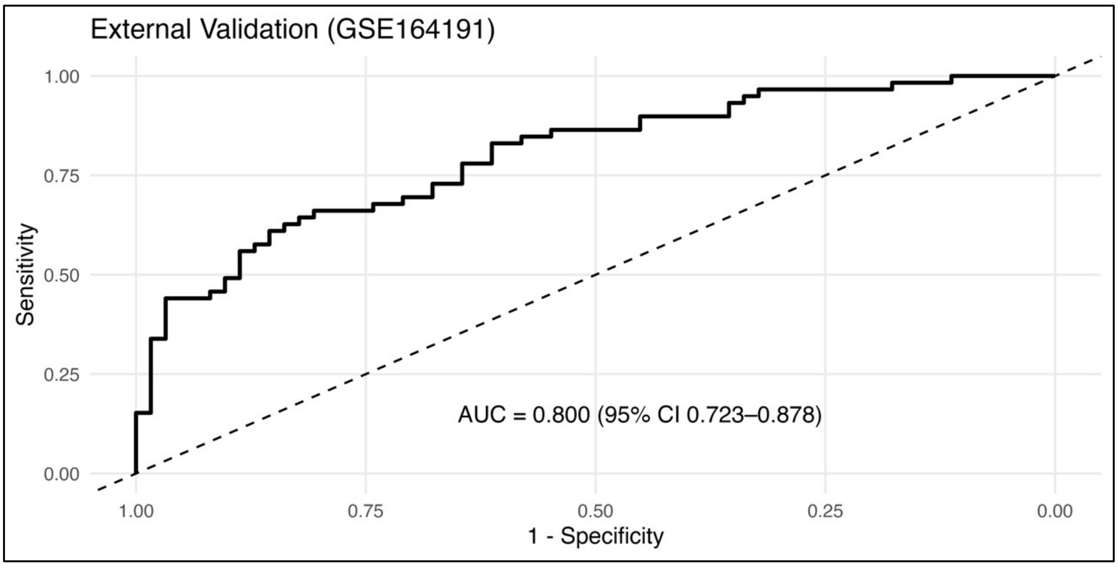

Supplementary Figure S3. External validation of the five-gene logistic regression model in the GSE164191 cohort. ROC curve showing the diagnostic performance of the five-gene whole-blood signature in an independent RNA-seq dataset (GSE164191). The solid line represents the fitted ROC curve, and the diagonal dashed line indicates the performance of a random classifier. The area under the curve (AUC) and its 95% confidence interval are displayed within the panel.

Supplementary Table S2. Diagnostic performance comparison of individual transcripts and the multigene logistic regression model in the RT-qPCR validation cohort.

Model	Included markers	AUC	95% CI	Sens Sp=90%	Threshold	Sens Sp=95%	Threshold
DLG5 (single-gene)	DLG5	0.758	0.524–0.993	0.667	0.301	0.556	0.318
CD177 (single-gene)	CD177	0.841	0.640–1.000	0.778	0.374	0.556	0.419
SH2D1B (single-gene)	SH2D1B	0.546	0.271–0.820	0.333	0.455	0.333	0.463
NQO2 (single-gene)	NQO2	0.179	0.000–0.365	0.000	0.482	0.000	0.492
KRT73 (single-gene)	KRT73	0.444	0.208–0.681	0.111	0.425	0.111	0.426
LR-5gene + Age/Sex	5-gene panel	0.952	0.884–1.000	0.889	0.410	0.667	0.583
LR-5gene (Stage I–II)	5-gene panel	0.929	0.868–0.989	0.839	0.284	0.839	0.394
LR-5gene (Stage III–IV)	5-gene panel	0.821	0.590–1.000	0.778	0.284	0.778	0.396

Abbreviations: AUC, area under the ROC curve; CI, confidence interval; Sens, sensitivity; Sp, specificity; Thr, threshold; CRC, colorectal cancer; HC, healthy controls; LR-5gene, multivariable logistic regression model based on the five-gene panel. Sensitivity was calculated at fixed specificity levels of 90% and 95%.

Supplementary Table S3. Summary of consistency across datasets

Gene	WB-PADs	WB-K	RT-qPCR validation	Consistency
**CD177**	↑	↑	↑	✓
**NQO2**	↑	↑	↑	✓
**KRT73**	↓	↓	ns	ns
**DLG5**	↓	↓	↑	Trend reversal
**SH2D1B**	↓	↓	↑	Trend reversal
